# Neuroimmune regulation in Hirschsprung’s disease associated enterocolitis

**DOI:** 10.3389/fimmu.2023.1127375

**Published:** 2023-04-17

**Authors:** Haosen Ji, Dengming Lai, Jinfa Tou

**Affiliations:** Department of Neonatal Surgery, Children’s Hospital, Zhejiang University School of Medicine, National Clinical Research Center for Child Health, Hangzhou, China

**Keywords:** Hirschsprung’s disease enterocolitis, neuron, immunity, neonatology, gastroenterology

## Abstract

Neuroimmune pathways are important part of the regulation of inflammatory response. Nerve cells regulate the functions of various immune cells through neurotransmitters, and then participate in the inflammatory immune response. Hirschsprung’s disease (HD) is a congenital abnormal development of intestinal neurons, and Hirschsprung-associated enterocolitis (HAEC) is a common complication, which seriously affects the quality of life and even endangers the lives of children. Neuroimmune regulation mediates the occurrence and development of enteritis, which is an important mechanism. However, there is a lack of review on the role of Neuroimmune regulation in enterocolitis associated with Hirschsprung’s disease. Therefore, this paper summarizes the characteristics of the interaction between intestinal nerve cells and immune cells, reviews the neuroimmune regulation mechanism of Hirschsprung’s disease associated enterocolitis (HAEC), and looks forward to the potential clinical application value.

## 1 Introduction

Neuroimmune regulation is an important link to maintain the homeostasis of the body, and plays a key regulatory role in inflammation, tumor and other diseases. Neuroimmune pathways are involved in the regulation of intestinal inflammation. On the one hand, the nervous system regulates the function of immune cells through neurotransmitters or neuropeptides, while on the other hand, immune cells play a key role in neuronal injury, repair and differentiation. Single-cell RNA sequencing of human and mouse intestinal nervous system (ENS) components shows that healthy intestinal neurons express soluble mediators and cell surface molecules and can communicate with innate and adaptive immune cell types (1). The activation of ENS induced by infection or inflammation can inhibit the progression of inflammation and restrict the pathological process, while the destruction of ENS structure may further aggravate inflammation and infection (2–4). During intestinal inflammation, the vagus nerve and its neurotransmitter can reduce enteritis in mice (5), and muscular macrophages limit neuronal damage through epinephrine signaling pathways (6). In many cases, recovery is limited, and damage to ENS may have long-term consequences, for example, gastrointestinal diseases after infection (7). This article reviews the research progress of intestinal nerve-immune cell interaction in intestinal inflammation.

## 2 Regulation of intestinal nervous system on immune cells

ENS is the largest component of the peripheral nervous system, which consists of two cell types: neurons and enteric glial cells (EGCs). EGCs support the ENS network and maintain the integrity of the epithelial barrier.

Intestinal neurons work by secreting neurotransmitters and neuropeptides. ENS participates in the maintenance of intestinal homeostasis in intestinal physiology. Intestinal submucosa and myenteric plexus are involved in regulating immune response, killing or excreting pathogens, and restoring mucosal barrier during enteritis. Intestinal nervous system is indispensable for the recovery of intestinal homeostasis.

### 2.1 Enteric neurons and intestinal immune cells

#### 2.1.1 Adrenergic neuronal regulation of macrophages

In the intestinal myenteric plexus, the myenteric macrophages (MMs) are closely connected with the cell bodies of excitatory endogenous enteric neurons (8). MMs are the main source of bone morphogenetic protein 2 (BMP2), which stimulate intestinal neurons to regulate gastrointestinal motility. In turn, the development and reproduction of MMs are controlled by colony stimulating factor (CSF1) expressed by intestinal neurons (9). In the intestine of CSF^−/−^ mice, the number of NO^+^ neurons increases, and there is no expression of bone morphogenetic protein 2 in MMs, which leads to the immature development of neurons in the intestinal muscle layer. Macrophage depletion induced by anti-CSF1R treatment affects the differentiation of Paneth and other intestinal epithelial cells (10).

After intestinal bacterial infection, MMs is activated, which plays an important role in the protection of intestinal neurons. This is attributed to the rapid activation of external sympathetic neurons innervating the intestinal muscle layer, and the NE secreted by norepinephrine neurons binds to the β2-adrenergic receptor(β2AR) on MMs.

NE/β2 signal in MMs induces the expression of arginase-1 *in vitro* (8). Arginase 1 (Arg-1) mediates the production of neuroprotective polyamines, for example, spermine (11), Spermine can inhibit the activation of NLRP6 inflammatory bodies and inhibit NLRP6-Caspase-11-mediated neuronal injury (12). When spermine and DFMO (an inhibitor of spermine synthesis) were added to drinking water, it was found that the damage to intestinal neurons increased in the inhibition group. The protective effect of Arg-1 on neurons after intestinal infection was proved by hybridization between LysM^Cre^ mice and Arg-1^flox/flox^ mice (13). The macrophages of this selective activation program play an important role in the repair and protection of intestinal tissue, and this discovery lays a foundation for exploring MMs as a neuroprotective sentinel.

Furthermore, the effect of adrenergic neurons on MMs plays a central role in coordinating the tissue protective tolerance response after Salmonella enteric infection. It has been found that wild-type mice infected with an attenuated Salmonella typhimurium (spiB) can clear the infection within 7-10 days, but the result of infection is long-term (up to 4 months) impairment of gastrointestinal motility and reduction of intestinal neurons in a NLRP6/Caspase11-dependent manner. MMs deletion or impaired NE/β2 signaling can worsen spiB infection-induced enteric neuronal injury. Exogenous β2 receptor agonists enhance Arg-1 expression in MMs, protect enteric neurons, and limit the effects of spiB infection on gastrointestinal motility (13). One experiment used mice infected with S. commissioneri roundworms (Sv) followed by Salmonella infection. Mice showed reduced intestinal nerve loss compared to Salmonella infection alone. This suggests that the adrenergic signaling pathways in myeloid macrophages limit infection-induced neuronal loss by way of immune tolerance (6).

#### 2.1.2 Cholinergic neuronal regulation of macrophages

In a model of postoperative intestinal obstruction in mice and humans (characterized by a surgically induced inflammatory response in intestinal myenteric macrophages and impaired intestinal motility), vagal stimulation or pharmacological action on cholinergic enteric neurons prevented myenteric macrophage activation and reduced postoperative intestinal obstruction in a α7 nicotinic receptor (α7nAChR) dependent manner (14). In a mouse model of food allergy, vagus nerve stimulation ameliorates intestinal inflammation caused by food allergy in a 7nAChR-independent manner. This effect may be mediated by the vagal system through increased phagocytosis of allergenic substances by CX3CR1^+^ macrophages (15). Vagus nerve stimulation increased the phagocytic activity of CX3CR1+ macrophages, an effect that may be dependent on 4b2nAChR. Food antigen uptake and subsequent induction of food-specific Treg by anti-inflammatory CX3CR1+ macrophages is effective in preventing allergic reactions (16, 17).

The ganglion-free colon of HSCR patients lacked intrinsic myenteric and submucosal plexuses, but cholinergic nerve fibers were significantly thickened, and the incidence and prognostic level of postoperative HAEC correlated with the proliferation of cholinergic nerve fibers. Some researchers divided the intestinal mucosa into high-fiber tissue and low-fiber tissue according to the degree of fibrous hyperplasia, and by immunofluorescence co-localization they found that macrophages from high-fiber tissue were associated with Tubulin+AChE+ neurons, and macrophages from the mucosal lamina propria of ganglion descending colon tissue from HSCR and control patients appeared to be associated with Tubulin+AChE- neurons. The physical proximity of cholinergic neurons to macrophages facilitates the interaction between the two (18). Analysis of the transcriptome of myeloid macrophages from high-fiber tissue and low-fiber tissue corresponding colonic segments, they found a trend of elevated levels of nicotinic and muscarinic receptors, high expression of CX3CR1, CD14+ (M2 macrophage marker) in macrophages isolated from high-fiber tissue. The percentage of Treg cells was significantly elevated. Macrophages exhibited an anti-inflammatory, tissue-resident phenotype. In contrast, in low-fiber tissue, the expression of cellular inflammatory factors such as IL-17, IL-6, and IL-1b was significantly increased, CCR2+ was highly expressed in macrophages, and the ratio of Th17/Treg was significantly elevated (18).

Under inflammatory conditions, monocyte-derived CCR2+ macrophages infiltrate the intestinal mucosa and initiate Th17 cell responses. Under steady-state conditions, CX3CR1^+^ macrophages secrete anti-inflammatory cytokines and maintain Treg numbers. CX3CR1^+^ macrophages are essential for epithelial cell integrity, control of bacterial translocation, and commensal tolerance (16). Muscarinic acetylcholine receptor activates ALDH gene expression in macrophages (antigen presenting cells) of CX3CR1+, which produces retinoic acid and promotes the expansion of intestinal Treg cells (19). In addition, Treg cells are able to inhibit the action of Th17 cells (20), Proliferative cholinergic nerve fibers influence the differentiation of CD4+ helper cells, especially the proportion of Treg, through their regulatory effects on macrophages, thereby regulating intestinal inflammation.

In the follow-up of 42 HSCR patients 1 year after surgery, 9 patients developed HAEC, of which 7 showed a low-fiber phenotype and 2 showed a high-fiber phenotype; these observations suggest that HSCR patients with a low-fiber phenotype are at a higher risk of developing HAEC after surgery (18), This study identified and explained the relationship between the high-fiber phenotype of the intestinal mucosa and the incidence of HAEC after the surgery of HSCR from an immunological perspective, providing a theoretical basis for the prophylactic treatment of HAEC.

In the further, intestinal serotonergic neurons are also cholinergic neurons. Tryptophan hydroxylase 2 (THP2), a marker of 5-HT expression in serotonergic neurons, is significantly decreased in intestinal neurons of HAEC patients and is restored postoperatively. Although only 5% of intestinal 5-HT is stored in neurons, only neuronal-derived 5-HT plays a role in regulating intestinal motility compared to intestinal epithelial-derived 5-HT. HAEC-mediated 5-hydroxytryptaminergic neuronal damage may lead to colonic dysfunction and recurrent enterocolitis (21). Another study shows that cholinergic innervation in rectosigmoid colon in HSCR patients is associated with secretion of pro-inflammatory IL-8, which increases the risk of HAEC (22).

Patients with HSCR typically present with exogenous cholinergic nerve fibers throughout the aganglionic rectosigmoid. Studies have shown that cholinergic signaling can reduce inflammatory responses and α7nAChR plays an important role in the neuroimmune signaling pathways. Thus, sparse exogenous cholinergic innervation in the sigmoid mucosa and antagonism of α7nAChR are associated with increased frequency of inflammatory immune cells and higher incidence of HAEC in patients with HSCR (22).

#### 2.1.3 Neuronal regulation of lymphocytes

Neurokinin P is also called substance P (SP). Endocrine neurons are the main source of intestinal SP. SP acts through interaction with cell surface receptors NK1R, NK2R and NK3R. SP induces peripheral blood mononuclear cells (including lymphocyte proliferation and immunoglobulin production) to stimulate the production of pro-inflammatory cytokines such as IL-1β, IL-6 and TNF-α (23–25). This may be due to the positive regulation of NK1R, as inflammatory factors such as IL-12, IL-18 and TNF-α induce NK1R expression in T cells (26), while IL-10 and TGF-β decreased the expression of NK1R (27). NK1R has the greatest affinity for SP, which is primarily associated with inflammatory processes (28). NK1R receptor density is significantly increased in patients with Crohn’s disease and ulcerative colitis (29). Expression of substance P was also shown to correlate directly with the severity of Trypanosoma cruzi megacolon. It was higher in submucosal and myenteric plexus neurons in the dilated portion of the megacolon compared to the undilated portion and the uninfected population, and this might be related to the preferential destruction of inhibitory motor neurons (VIP and NOS immunoreactivity) in the intestine of chagasic patients with megacolon by Trypanosoma cruzi and inflammatory processes (30). However, another study described lower myenteric and submucosal plexus SP concentrations in rectal samples from trypanosomatid patients, which may be associated with reduced intestinal peptidergic neuronal damage (31).

VIP^+^ neurons mediate VIP secretion, and VIP not only monitors intestinal epithelial status by regulating lymphocytes, but also regulates mononuclear phagocytes (MNPs) and the cytokines they secrete, tilting immunity toward the Th2 type (32). VIP-sensitized dendritic cells induce Treg production and restore immune tolerance. VIP-producing neurons in the intrinsic layer are very close to ILC3, which selectively expresses type 2 VIP receptors.IL-22 secretion is inhibited by ILC3 binding to VIP, which results in reduced levels of AMP, an epithelial-derived antimicrobial peptide, and rhythmic changes in VIP levels promote normal function of the intestinal barrier (33).

Using a mouse model of intestinal helminth infection, it has been shown that neuromedin U increases protective immune responses and induces parasite clearance through activation of ILC2 (34, 35). Conversely, signals from sympathetic adrenergic neurons attenuate diminished ILC2 effector function and prevent chronic pathological type 2 inflammation by activating ILC2-specific β2-adrenergic receptors (36).

Enteric-specific IL-18 neurons drive the production of the antimicrobial peptide AMP in goblet cells and protect the intestine during Salmonella typhimurium infection, which is important for the mucosal barrier and coordinated homeostasis of the intestine (37).

Neuropeptides have innate host defense enhancing and direct antimicrobial effects and some neurons distributed in the intestine are one of the main sources. Neuropeptides regulate intestinal immunity in endocrine and paracrine ways. In patients with sepsis, the intestinal barrier is damaged, intestinal permeability is increased, and bacterial cell wall components (e.g. lipopolysaccharide) can stimulate nociceptive receptors located in the lamina propria of the intestine and induce increased synthesis and release of calcitonin gene related peptide (CGRP), substance P, neuropeptide Y, vasoactive intestinal peptide, VIP and other neuropeptides. CGRP can directly act on lymphocytes, dendritic cells and macrophages in the intestinal tract, significantly improving the host’s intestinal defense ability (38).

#### 2.1.4 Neuronal regulation of mast cells

The release of SP from peptidergic neurons activates mast cells and promotes their degranulation, followed by the release of pro-inflammatory mediators from mast cells, such as TNF-α, IL-1β and trypsin. They strengthen the relationship among mast cells, trypsin, neuroinflammation and neuronal death (39–41). After co-culture of intestinal secretory neurons with mast cells, mast cells release histamine and protease through degranulation resulting in increased intra-neuronal Ca2+ concentration and significantly increased neuronal sensitivity, but the function of mast cells themselves is inhibited (42).

### 2.2 Relationship between enteric glial cells and immune cells

EGCs are distributed in different levels of the intestine and show the phenotype of GFAP^+^ after activation. EGCs can be divided into two subgroups based on the expression of GFAP (43). LPS and proinflammatory cytokines can induce the expression of GFAP (44), and the combination of LPS and IFN-y or high concentration of IL-10 (5-100ng/ml) can induce the proliferation of enteric glial cells (45, 46). However, IL-1 β or low level of IL-10 (0.1ng/ml) can prevent the proliferation of ECGs (46). The expression of GFAP has been proved to be related to neuronal injury or protection and inflammation. Glial cells of GFAP^+^ secrete, which directly and positively affects the growth, maturation and survival of neurons (44). GDNF can inhibit the activity of MPO, the expression of IL-1 β and TNF- α, and increase the expression of ZO-1 and Akt. It is directly involved in the restoration of epithelial barrier function *in vivo* by reducing the increase of epithelial permeability and inhibiting mucosal inflammation. GDNF strongly prevents apoptosis and significantly improves DSS experimental colitis *in vivo* (47). An experimental model of intestinal inflammation has shown that the lack of glial cells will lead to severe tissue inflammation and intestinal necrosis after ablation of enteric glial cells of transgenic mice with ganciclovir (48). The factors produced by EGCs have strong anti-apoptotic activity, protect IEC barrier and maintain the permeability of intestinal barrier (49, 50).

Interestingly, another experiment showed that ECGs stimulated by LPS and IFN-y accelerated proliferation and produced inflammatory mediator iNOS, which aggravated intestinal inflammation (45). In patients with inflammatory bowel disease, the proportion of GFAP^+^ in EGCs is increased, and its secreted mediators increase the permeability of intestinal mucosal barrier, thus aggravating inflammation. But in normal people, the medium secreted by EGCs has a protective effect on intestinal mucosa (51). ENS undergoes structural and phenotypic plastic changes during inflammation. It is not only affected by inflammation, but also actively participates in the inflammatory process (52) Gut-associated neurons are closely related to immune cells. They continuously monitor and regulate intestinal function, including intestinal motility and nutrition perception, and maintain homeostasis (13).

#### 2.2.1 Enteric glial cells and lymphocytes

In a study using RET deficient mice, the authors demonstrated that microorganism-induced glial cell-derived GDNF (ligand) could can activate intestinal ILC3 subsets to express neuroregulatory receptor tyrosine kinase (RET) (53).Activated RET could induce IL3 to secrete IL-22 and increase the expression of tight junction proteins in epithelial cells, which could effectively promote intestinal homeostasis (53). When EGCs are exposed to pro-inflammatory cytokines (IL-1 β and/or TNF-α), IL-7 can also be up-regulated. T lymphocyte function analysis shows that the induced expression of classical IL-7 can protect T cells from cell death (54). In patients with megacolon caused by Trypanosoma cruzi, EGCs express II-type HLA-DR and B7 costimulatory molecules. Therefore, EGCs also act as an antigen-presenting cell on the activation of lymphocytes, thus affecting the progress of chronic digestive trypanosomiasis (55).

#### 2.2.2 Enteric glial cells and macrophages

Pro-inflammatory signal pathways induce Cx43-dependent M-CSF production in glial cells through protein kinase C (PKC) and tumor necrosis factor invertase (TACE), and M-CSF promotes the transformation of intestinal myometrial macrophages to M1. Connexin-43 is needed for communication between glial cells in this process. Knockout of Connexin-43 in glial cells can prevent the development of visceral hypersensitivity after chronic colitis (43).

#### 2.2.3 Enteric glial cells and mast cells

The interaction between EGCs and mast cells may inhibit intestinal inflammation. The activation of mast cells can promote the activation of enteric glial cells and macrophages, resulting in intestinal mucosal injury and neuronal reduction (56). GDNF from EGCs can also significantly inhibit mast cell degranulation and reduce inflammation (57).

## 3 Regulation of intestinal immune cells on nerve cells

### 3.1 Regulation of neurons by lymphocytes

Intestinal lymphocytes are mainly divided into T lymphocytes, ILC, B lymphocytes and so on. Among T cells, CD4+T lymphocytes are helper T cells (Th), which play a major role in cellular immunity. CD8+T lymphocytes are cytotoxic T cells (Tc), which are a kind of effector cells with killing activity. The CD4+ cells that play an important role in intestinal inflammation are Th1, Th2, Th17 and Treg. Th1 mainly secretes IFN-y and its function is to activate macrophages. Th2 mainly secretes IL-2, IL-4 and IL-5, which plays a major role in type 2 immunity (58). Th17 mainly secretes IL-6, IL-21 and IL-23, and its main role is to activate neutrophils and induce the production of antimicrobial peptides and tight junction proteins by intestinal epithelial cells, thus maintaining the integrity of the intestinal barrier. It is at the same time involved in inflammation and autoimmune diseases and has a powerful pro-inflammatory function (58–60). Treg cells regulate T-cell function and prevent autoimmunity by producing the anti-inflammatory cytokines IL-10, IL-2, and TGF-β. Their dysregulation is associated with several inflammatory and autoimmune diseases (58, 61). Th17 cells and Treg cells are two important subsets of lymphocytes with opposite functions. Despite having different functional properties, differentiation from naive T cells to Th17 cells and Treg cells is dependent on the expression level of TGF-β (62), The delicate balance between Th17 cells and Treg cells is the key to the internal and external environment of gastrointestinal tract (63).

Innate lymphoid cells (ILCs) can be divided into four types: type I innate lymphoid cells, ILC1; type II innate lymphoid cells, ILC2; type III innate lymphoid cells, ILC3 (64); regulatory innate lymphoid cells (ILCregs). The main cytokines secreted by ILC1, ILC2, ILC3 and ILCregs are similar to those secreted by Th1, Th2, Th17 and Tregs (65).

B lymphocytes play an immunoprotective role in the gut by producing IgA antibodies. Secreted into the intestinal lumen, SIgA binds to gut microbes and food antigens, which avoids potentially harmful stimulation of the mucosal immune system by lumen contents, and it also helps regulate the composition of the microbiome (66). The potential role of IgA in the pathogenesis of HAEC was first demonstrated by Iamura et al. They found that IgA containing plasma cells were significantly increased in the lamina propria along the entire length of resected bowel in enterocolitis patients, compared with non-enterocolitis patients. They also found decreased luminal IgA (sIgA) in the same patients (67). These results were verified in the animal model of HAEC in another research. Moreover, they noted small intestinal lamina propria pIgR, which transports and secretes IgA into the lumen, was decreased ∼50% in EdnrBNCC^−/−^ mice (68). In conclusion, these results suggest a decrease in IgA production or transport in HSCR and HAEC.

It has been observed in animal models of Parkinson’s that CD4+ T cells drive inflammatory responses in the intestinal mucosa and reduce the number of dopaminergic neurons in the myenteric and submucosal plexuses (69). Th2 is a major source of IL-4, while IL-4 and IL-13 together coordinate alternative activation of macrophages, including upregulation of Arg-1, which may play a protective role against neurons during intestinal infections (70). In parasite-associated colitis involving Th2-type T cells, cytokines such as IL-4 and IL-13 promote smooth muscle contraction by activating the STAT6 pathway on the one hand, and on the other hand, mast cells are recruited by IL-4, and mast cell degranulation further induces neuronal hyperresponsiveness thereby promoting intestinal smooth muscle contraction and ultimately intestinal parasite expulsion. In contrast, colitis, in which Th1-type cells play a dominant role, results in inadequate muscle contraction (71).

### 3.2 Regulation of neurons by mast cells

Mast cells are granulocytes that contain histamine, 5-hydroxytryptamine and other inflammatory mediators, which are released by cell disintegration and can cause inflammation in the tissue (72). Mast cells are located near blood vessels and nerve fibers and can act bidirectionally with the nervous system, which makes them ideal candidates for modulating neural activity and nociception; trypsin and histamine released from mast cells can cause the release of neuropeptides, and substance P(SP) and calcitonin-related peptides released from proximal nerve endings can further activate mast cells (73). Neuronal hyper-reactivity is a result of MC degranulation, which can lead to excessive gastrointestinal secretion, resulting in diarrhea, abdominal pain and cramps (74).

Trypsin and chymotrypsin are the major serine proteases secreted by mast cells, and an increase in protease-secreting mast cells correlates with a decrease in PGP9.5-associated neurons in the megacolon (75). In addition, only the increase in trypsin-related mast cells was associated with a decrease in PAR2-IR neurons (protease-activated receptor 2-associated neurons), which are cleaved by trypsin and involved in neuronal death, triggering alterations in chronic intestinal function (76). Elevated expression of PAR-1 and PAR-2 in the colon of HSCR patients suggests that localized excessive release of PAR-activated proteases may trigger an inflammatory response, leading to HAEC (77). Therefore, it is likely that mast cells are involved in the process of HAEC development. Other mediators secreted by mast cells such as IL-6 and prostaglandin 2 can also induce neuronal death (78).

HSCR is a rare congenital disorder caused by the absence of ganglia in the submucosa and myenteric plexus of the colon. In the absence of neuron in the intestinal wall, hypertrophic nerve trunks are associated with an increased number of adrenergic and cholinergic nerve fibers (79–81). The number of mast cells in the aganglion segment of the colon in HSCR patients was significantly higher than in the walled segment with neurons, and the mast cells in the intestinal segment showed a transmural distribution. Some mast cells, which are in direct contact with the mast nerve trunk of aganglion segments, can synthesize, store and release neurotrophic factors that support nerve fiber development and maintenance, suggesting that they may be essential for nerve growth and repair (80–82).

### 3.3 Regulation of neurons by macrophages

Macrophages are an important part of the body’s intrinsic immune system and are widely distributed in various tissues and organs, with the functions of phagocytosis, antigen presentation and secretion of various cytokines, playing an important role in physiological processes such as inflammation, defense, repair and metabolism. According to the location of macrophages in the intestine, they are divided into MMs and lamina propria macrophages (LPMs). MMs exhibit a tissue protective phenotype (8). MMs interact with neurons of the myenteric plexus and present as a bipolar shape (9). MMs highly express CX3CR1 and CD14 (an M2 macrophage marker) (18), and CX3CR1+ macrophages are the major antigen-presenting cells, which are essential for the differentiation of CD4^+^ helper T cells, especially for the cell proliferation and maintenance of Treg (83). Transcriptional profiling revealed that MMs express genes associated with anti-inflammatory activity (Retlna, Mrc1, CD163, IL-10) in the activated state, while mucosal LPMs preferentially express the pro-inflammatory phenotype (8). In the absence of infection, MMs secrete BMP2 in a microbial-dependent manner to regulate neuronal activity and intestinal motility (9), and the expression of myeloid macrophage protective gene profile is enhanced after infection (8). *In vitro*, after macrophages were polarized to M1 type by LPS, pro-inflammatory cytokines (IL-1β, IL-6 and TNF-α) contained in macrophage supernatants inhibited the expression of OT and OTR in intestinal neurons; after macrophages were polarized to M2 type by IL4, anti-inflammatory cytokines (TGF-β) contained in macrophage supernatants promoted OT and OTR in intestinal neuronal expression. Therefore, it has been suggested that macrophages are polarized to M1 type during the inflammatory phase and to M2 type during the recovery process (84).

## 4 Interstitial cells of Cajal and macrophages

Interstitial cells of Cajal(ICCs) are closely related to the intestinal nervous system and distributed in a reticular pattern between the intestinal plexus and smooth muscle (85). ICCs are pacemakers of gastrointestinal slow wave and mediators of enteric motor neurotransmission. There are many kinds of neurotransmitter receptors on the surface. Intestinal nerves release neurotransmitters to regulate the frequency of slow wave generated by ICCs, thereby regulating intestinal motility (86). The function of ICCs plays an important role in HSCR and HAEC. Through the study of sigmoid colon from patients with Hirschsprung’s disease in 2008, it was found that the nubmer of ICCs expressing c-kit decreased significantly, while the number of ICCs expressing CD34 did not decrease, so it was pointed out that the specific downregulation of c-kit in ICCs may be a cause of sigmoid megacolon (87). Interestingly, tumor necrosis factor-α secreted by colonic M1 macrophages can result in intestinal dysmotility in HAEC by causing interstitial cells of Cajal (ICCs) to lose their c-kit phenotype and impair their pacemaker function through NF-κ B/miR-221 pathway (88). The loss of ICCs function will aggravate the disturbance of intestinal motility, resulting in the accumulation of intestinal contents, damage of intestinal mucosal barrier, invasion of LPS and increase of M1 macrophages.

## 5 Intestinal neuroimmune modulation in the diagnosis and treatment of HAEC

The interaction between nerve cells and Immune cells(e.g. mast cells) plays an important role in HAEC (82). Compared with HSCR intestinal segment, exogenous cholinergic fibers decreased and M1 macrophages increased in HAEC intestinal segment (22, 88). There have been many diagnostic techniques and treatments based on the discovery in clinic. Sodium cromoglycate (SCG) is a non-absorbable mast cell stabilizer with no systemic side effects and is effective in treating chronic or recurrent colitis in patients with congenital megacolon (89). In addition, it has been found that the size of the muscle unit/neuron ratio can be used to determine the transition zone of long-segment congenital megacolon, which can better determine the area of surgical resection. The type and content of mucosal nerve fibers and the size of the intestinal ganglion in the intraoperatively resected intestine can also be used as predictors to determine the probability of enterocolitis after HSCR (18, 90).

**Figure 1:**
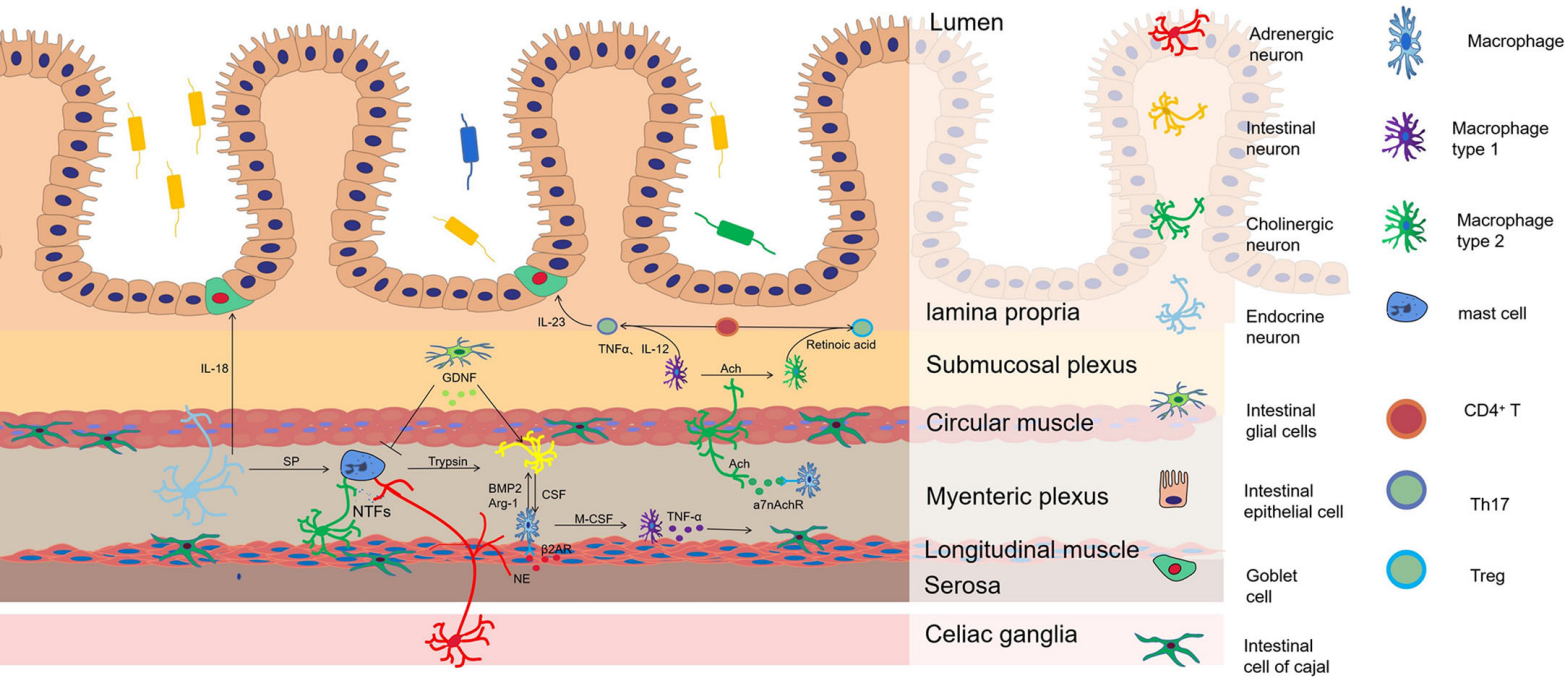
The mechanism of neuroimmune regulation in HAEC. Macrophages are the main source of bone morphogenetic protein 2 (BMP2), which stimulate intestinal neurons to regulate gastrointestinal motility. In turn, the development and reproduction of Macrophages are controlled by colony stimulating factor (CSF1) expressed by intestinal neurons. Adrenergic nerve fibers regulate muscular macrophages to participate in the protection of neurons by acting on β2AR. Cholinergic nerve fibers regulate the conversion of macrophages from M1 to M2 to inhibit the development of inflammation by acting on a7nAchR, which increases the ratio of Treg/Th17 in intestinal mucosa. M1 macrophages release TNF-α that makes cells of Cajal lose the c-kit phenotype and causes intestinal motility disorder. In the aganglionic segment of colon in patient with HSCR, mast cells release neurotrophic factors (NTFs) to promote the proliferation and hypertrophy of nerve fibers. SP from peptidergic neurons promotes mast cells degranulation, resulting in releasing of pro-inflammatory mediators such as TNF-α, IL-1β and trypsin, inducing neuronal injury. GDNF from EGCs can significantly inhibit mast cell degranulation and reduce inflammation. A sharp arrow indicates functional promotion, while a flat arrow indicates inhibition.

Crohn’s disease and HAEC have somewhat similar pathogenetic processes. Both have impaired nutrient absorption due to intestinal obstruction, decreased immune defense due to disruption of the intestinal mucosal barrier, and abnormal immune responses due to disturbances in the intestinal flora; therefore, it is likely that both share a neuroimmune regulatory mechanism that is given in common (**Figure 1**). In clinical studies, stimulation of the vagus nerve was found to reduce intestinal inflammation (91). Vagal tone is reduced in patients with Crohn’s disease, and stimulation of the vagus nerve can be effective in treating active Crohn’s disease (92). Abdominal vagus nerve stimulation improves postoperative bowel obstruction (93). All of the above clinical studies suggest that stimulation of the vagus nerve may be an effective way to prevent and treat HAEC.

## 6 Prospect

The loss of neurons in the distal colon makes the colons of patients with HSCR in a unique neuroimmune regulation environment. Significant progress has been made in understanding the mechanisms of HSCR and HAEC, but there is still a lot to be explored about the neuroimmune regulation of HAEC. The study of intestinal neuro-immune cell interactions can open up new ideas for the treatment of HAEC. In terms of immune cell regulation, treatment targeting macrophages or ICCs may represent promising therapeutics (88). Targeted therapies against IL-8 (22) and against pIgR-mediated sIgA translocation may be effective in treating HSCR-induced inflammation (68). The modulation of mast cell function is expected to reshape the neural distribution in the HSCR gut and enhance the neuromodulatory effect, thus preventing HAEC. In the regulation of neurotransmitters, neuropeptides such as substance P, neuropeptide Y and vasoactive intestinal peptide carry a cationic charge and can directly break the membrane to kill bacteria, which is less likely to produce resistance than traditional antibiotics (94). These may provide new ideas for anti-infective treatment of sepsis due to severe HAEC. There are various ways to activate vagal pathways to reduce inflammation, including direct stimulation by physical means and the use of 5-HT agonists, and therapeutic modalities derived from this have greater promise for clinical application. For future studies, the application of human brain neural interfaces may enhance gut neuroimmune regulation in HSCR patients. In the field of basic medicine, further studies are needed to elucidate whether immune cells under the influence of specific neurotransmitters are innervated by the corresponding neurons and the effects they produce under healthy and disease conditions, and the study of this process will provide a theoretical basis for achieving precise neuroimmune regulation.

## Author contributions

HJ draft the article and revised it critically, DL gave the original idea and design, JT confirmed the final release. All authors contributed to the article and approved the submitted version.
